# Isothiocyanates as Multi-Target Natural Compounds in Leukemia: Mechanisms, Selectivity, and Therapeutic Potential

**DOI:** 10.3390/ijms27125620

**Published:** 2026-06-22

**Authors:** Alberto Yoldi Vergara, Kristina Simonicova, Anna Bertova, Zdena Sulova, Albert Breier, Denisa Imrichova

**Affiliations:** 1 Institute of Molecular Physiology and Genetics, Centre of Biosciences, Slovak Academy of Sciences, Dúbravská Cesta 9, 841 04 Bratislava, Slovakia; umfgyolv@savba.sk (A.Y.V.); kristina.simonicova@savba.sk (K.S.); anna.bertova@savba.sk (A.B.); zdena.sulova@savba.sk (Z.S.); 2 Institute of Biochemistry and Microbiology, Faculty of Chemical and Food Technology, Slovak University of Technology in Bratislava, Radlinského 9, 812 37 Bratislava, Slovakia

**Keywords:** isothiocyanates, leukemia, resistance, chemotherapy

## Abstract

Natural compounds are increasingly explored as complementary strategies to enhance the effectiveness of chemotherapy and reduce toxicity. Among these are isothiocyanates (ITCs), bioactive metabolites derived from glucosinolates in cruciferous vegetables, which have gained substantial attention for their chemopreventive and antileukemic potential. ITCs exert diverse biological effects driven by the high reactivity of the –NCS group, enabling covalent modification of key cellular proteins and modulation of signaling pathways. Well-studied representatives, including sulforaphane (SFN), allyl isothiocyanate (AITC), 6-(methylsulfinyl)hexyl isothiocyanate (6-MITC), benzyl isothiocyanate (BITC), and phenethyl isothiocyanate (PEITC), exhibit diverse antileukemic activities, including cytotoxic, pro-apoptotic, differentiation-inducing, and cell-cycle-modulating effects. Although individual compounds differ in their relative potency and predominant biological responses, their activities are generally mediated through multiple interconnected mechanisms including oxidative stress modulation, mitochondrial dysfunction, regulation of apoptosis-related proteins, and interference with key signaling pathways. In addition to apoptosis, several ITCs have also been reported to induce autophagy, ferroptosis, or cellular differentiation in leukemic cells. Taken together, the existing evidence highlights ITCs as promising candidates for leukemia chemoprevention or therapy, acting through multi-targeted mechanisms that may complement conventional treatment strategies. Further studies are needed to clarify their selectivity, mechanistic diversity, and translational potential.

## 1. Introduction

In recent years, research has increasingly focused on the potential of natural compounds to enhance the effectiveness of chemotherapy. The dual aim of this research is to improve medical outcomes and mitigate treatment-related adverse effects. Phytochemicals, bioactive molecules produced by plants, are the subject of extensive research due to their potential therapeutic applications in treating various diseases, including cancer [1]. Many widely used anticancer drugs, such as taxanes, vinca alkaloids, camptothecin derivatives, and podophyllotoxins, originate from plants [2]. In addition to their direct pharmacological effects, several plant molecules have been shown to possess chemopreventive properties. In other words, these substances can prevent the onset of disease or limit its progression. Chemoprevention is now widely accepted as a strategy for preventing various diseases. Historically, herbal medicines were used to treat many diseases, and their use has increased significantly with the introduction of bioactive substances into clinical practice. Their efficacy has been demonstrated through extensive in vitro and in vivo studies. The growing success of herbal medicines in the pharmaceutical market is due to their remarkable therapeutic efficacy and potent anticancer properties. Natural products derived from plants are a valuable source of biologically active compounds that can act as chemopreventive or chemotherapeutic agents. They exert anti-cancer effects by inhibiting cell proliferation, modulating the cell cycle, inducing DNA damage, suppressing DNA repair mechanisms, and disrupting key oncogenic signaling pathways.

**Figure 1:**
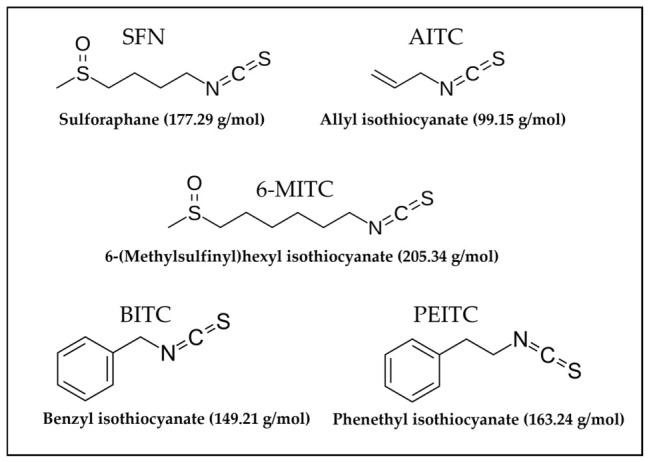
Chemical structures of the most common natural ITCs.

Isothiocyanates (ITCs) are aromatic and aliphatic compounds containing a reactive -NCS group. They are formed naturally through the enzymatic hydrolysis of plant glucosinolates (GSLs). Their antifungal activity [3] and cytotoxic effects on neoplastic cells [4,5] were first demonstrated in the late 1960s. Although the anticancer properties of ITCs were recognized over five decades ago, and supporting evidence has continued to accumulate, extensive and systematic research into these compounds remains ongoing [6,7]. The most common natural ITCs (Figure 1) are sulforaphane (SFN), allyl isothiocyanate (AITC), 6-(methylsulfinyl)hexyl isothiocyanate (6-MITC), benzyl isothiocyanate (BITC), and phenethyl isothiocyanate (PEITC). This article aims to compile and critically summarize the available information on the anticancer effects of natural ITCs and their derivatives, providing a contemporary overview of these substances.

## 2. Isothiocyanates—Chemical Reactivity and Biological Implications

ITCs are a diverse group of naturally occurring compounds. These include aliphatic (e.g., AITC and SFN) and aromatic (e.g., BITC and PEITC) types. ITCs can also be synthesized chemically. One of the most widely used synthetic ITCs is fluorescein isothiocyanate (FITC), which is primarily used for fluorescent labeling of proteins [8]. ITCs have been identified also as inhibitors of P-type ATPases. Compounds such as p-bromophenyl isothiocyanate, fluorescein isothiocyanate, and [4-isothiocyanatophenyl-(6-thioureidohexyl)-carbamoylmethyl]-ATP form covalent bonds with either thiol (–SH) or amino (–NH_2_) residues within or proximal to the ATP-binding site. A well-characterized example of this interaction is the plasma membrane sodium–potassium pump [9,10,11]. These compounds are well known for their wide range of biological activities [12,13]. These effects arise from the intrinsic reactivity of the ITC functional group (–NCS) and the physicochemical properties of the rest of the molecule, such as lipophilicity, geometry, molecular size, and rigidity. The reactivity of the –NCS group governs the ability of ITCs to form covalent bonds with the functional groups on small biomolecules or macromolecules. Meanwhile, physicochemical parameters largely determine their bioavailability and distribution across cellular and tissue compartments [14]. The physicochemical profile of a molecule, particularly its lipophilicity, is defined by the side chains carrying the –NCS group, whether aliphatic, aromatic, or a combination of the two. This profile influences its ability to penetrate cellular membranes and may modulate the magnitude of its interaction with the target structure [15].

The linkers (e.g., CH_2_ in BITC and C_2_H_4_ in PEITC) isolate the –NCS group from the aromatic ring’s delocalized π-electron system by connecting an aliphatic arm to the aromatic nucleus. This isolation influences the –NCS group’s reactivity. Such flexible linkers also facilitate the spatial orientation of –NCS groups during reactions by enabling free rotation around sp^3^-hybridized carbon atoms [16].

The –NCS group is highly susceptible to nucleophilic attack by functional groups containing heteroatoms (N, O, or S) with an unshared electron pair and a partial negative charge. The most common nucleophilic groups in amino acids, peptides, and proteins are –SH, –OH, and –NH_2_ [15]. When the reactivity of a primary –NH_2_ group towards ITCs is set to 1 by convention, the reactivity of -OH is approximately 0.2. In contrast, the reactivity of the –SH group ranges from 1000 to 1,000,000 [14,15]. The high reactivity of –SH groups arises from their acid–base properties. A proton (H^+^) can dissociate from them, leaving behind an S^−^ anion that has a strong affinity for the –NCS group. Unlike reactions involving –SH or –OH groups, which produce less stable S- or O-thiocarbamate esters, interactions between ITCs and –NH_2_ groups result in the formation of stable N, N′-disubstituted thiourea derivatives [15,16].

Although ITCs are often considered group-specific reagents that react randomly with nucleophilic sites on proteins, there is experimental evidence suggesting that selective modification of essential residues in enzyme active sites can occur. For example, the critical cysteine residue in the enzyme D-glyceraldehyde-3-phosphate dehydrogenase can be specifically modified by alkyl-, aryl-, and aralkyl-ITCs [17].

The biological activities of ITCs, driven by their chemical reactivity, have been the subject of intensive investigation over the past five decades. Recently, there has been a renewed scientific interest in naturally occurring ITCs from plant sources, largely due to accumulating evidence of their potential to prevent chronic diseases such as cancer, cardiovascular disease, neurodegenerative disease, and metabolic disorders [18]. However, some studies highlight the genotoxic potential of ITCs, which can either selectively target and destroy neoplastic cells or cause genetic damage in normal cells. In addition to their cytotoxic and genotoxic effects, ITCs can also influence the activity or function of various enzymes and proteins by covalently binding to biomacromolecules [19].

Several studies suggest that ITCs preferentially affect cells with high transcriptional and metabolic activity, which is accompanied by rapid proliferation, a feature characteristic of neoplastic transformation [20]. Consequently, numerous studies have demonstrated the effectiveness of natural ITCs in targeting cancer cell lines derived from various tissues, resulting in a significant reduction in their viability and proliferative capacity [21]. From a structure–activity relationship (SAR) perspective, the biological effects of individual ITCs are influenced not only by the conserved –NCS functional group but also by the chemical nature of the side chain. Differences in lipophilicity, steric properties, and linker length can affect cellular uptake, intracellular distribution, and target engagement. In general, aromatic ITCs such as BITC and PEITC often exhibit stronger cytotoxic activity than aliphatic ITCs, whereas compounds such as SFN are frequently associated with broader redox-modulating effects. However, these trends are highly context-dependent and may vary across cell types, experimental conditions, and intracellular redox status [22].

## 3. Cruciferous Vegetables and Chemoprevention: The Role of ITCs

Chemoprevention is a promising approach to inhibiting, delaying, or suppressing carcinogenesis by using naturally occurring or synthetic chemical agents, including ITCs [23]. The anticancer effects of ITCs affect different stages of the cancer process through various biological mechanisms. For example, ITCs can prevent carcinogens from binding to target cells, thereby affecting the initial stage of carcinogenesis. This can be achieved by increasing the detoxification of carcinogens, altering their uptake and metabolism, neutralizing reactive oxygen species (ROS) and other oxidative agents, and promoting DNA repair mechanisms [24,25]. Additionally, these compounds may inhibit the further development of cancer cells after initiation by influencing cell cycle regulation, signaling pathways, transcriptional mechanisms, and programmed cell death. This contributes to the suppression of tumor growth and spread. ITCs are sulfur-containing organic molecules found in high concentrations in cruciferous vegetables of the *Brassicaceae* family, such as watercress, broccoli, Brussels sprouts, cauliflower, and cabbage. These vegetables are a major source of ITCs in the human diet. The chemopreventive efficacy of ITCs is thought to be due to their ability to modulate multiple cellular pathways that reverse, inhibit, or delay carcinogenesis [18]. ITCs are formed by the enzymatic hydrolysis of GSLs, a reaction catalyzed by the plant enzyme myrosinase (β-thioglucoside glucohydrolase, EC 3.2.1.147). However, this occurs only when GSLs are consumed without prior heat treatment, such as cooking. When myrosinase is inactivated by high temperatures, however, GSLs are partially absorbed in the stomach and hydrolyzed in the large intestine by bacterial myrosinase [26].

The variety of ITCs that can be produced from naturally occurring GSLs is truly astounding. Their ability to reduce the risk of cancer or achieve a potential therapeutic effect varies considerably, partly because they affect different types of cancer in different ways. The chemoprotective and cytotoxic effects of these ITCs have been extensively studied, and their potential antineoplastic activity has been demonstrated in experimental models of cancer of the esophagus, mammary glands, lungs, livers, pancreas, colon, and small intestine, as well as in hematological malignancies [6].

## 4. Anticancer Potential of ITCs

A comparative study of four ITCs with well-established anticarcinogenic properties—AITC, BITC, PEITC, and SFN—revealed that their rapid growth-inhibitory effects were primarily dependent on the specific ITC rather than the cell type. However, the effectiveness also depended on the type of neoplastic cells and their genomic, epigenetic, transcriptional, and proteomic variations. Overall, the ITCs appeared more effective against hematological cancer cells than epithelial cancer cells [27]. Reported IC_50_ values of these ITCs in different leukemia cell lines and primary cells, collected from multiple published in vitro studies, are summarized in Table 1.

### 4.1. Variability in the Time-, Dose-, and Cell Line–Dependent Cytotoxicity of ITCs

#### 4.1.1. SFN

Sulforaphane is released by enzymes from glucoraphanin, the main GSL found in broccoli and other cruciferous vegetables. SFN is one of the most extensively studied ITCs to date. A growing number of human trials using SFN-rich preparations have been conducted across a range of conditions, including pilot studies in cancer patients. However, its definitive therapeutic efficacy remains to be established [45]. The effects of broccoli sprouts rich in SFN have been investigated in men with recurrent prostate cancer and in patients with advanced pancreatic cancer. Although treatment with SFN-rich extracts did not result in a > 50% decline in prostate-specific antigen (PSA) levels, it did significantly prolong PSA doubling time. The authors concluded that further studies are required to clarify the role of SFN as a preventive or therapeutic agent, including studies employing higher doses. Furthermore, studies on advanced pancreatic cancer highlight the necessity of clinical pilot trials to evaluate the efficacy of broccoli sprout extracts [46]. The aim of these studies is to assess the clinical feasibility and acceptability of broccoli sprout-based adjunctive therapy when combined with palliative chemotherapy. While these studies were conducted on solid tumors, they provide important translational evidence supporting the clinical relevance of SFN and other ITCs, thereby justifying further investigation of their anti-cancer potential in hematological malignancies.

To understand the potential cancer-preventive and anticancer effects of SFN, it is important to consider its multifaceted impact on metabolic and regulatory pathways, as well as on fundamental cellular balances and overall cellular homeostasis. The following text summarizes key findings obtained from leukemic cell lines and leukemia patient samples.

The IC_50_ for SFN varies significantly across studies and cell lines. In most cell lines, the IC_50_ decreased with prolonged cultivation time [40,42]. Similarly, comparing a three-hour SFN treatment followed by 69 h in SFN-free medium with a 72 h SFN treatment, the IC_50_ decreased by 5.7- to 11.2-fold [27]. However, the opposite trend was observed in a study by Núñez-Sánchez et al., in which the IC_50_ value doubled when comparing 48- and 72 h culturing [38].

Fimognari et al. demonstrated that SFN induces apoptosis in cells synchronized in the G1, S, and G2/M phases of the cell cycle. G1-phase cells were the most sensitive to SFN, followed by G2/M-phase cells, with S-phase-blocked cells the least sensitive. The authors concluded that this differential sensitivity is determined by the involvement of cell cycle-regulated proteins in SFN’s mechanism of action [47].

Several studies have shown that SFN arrests cells in the G2/M phase of the cell cycle and induces apoptosis in a time- and dose-dependent manner [28,32,39,42,48,49,50]. It has been associated with the activation of caspases [42,50,51,52], cleavage of PARP (Poly(ADP-ribose) polymerase) [42,51,52,53], upregulation of BAX (BCL2-associated X) [48,49,50], downregulation of Bcl-2 (B-cell lymphoma 2), increased release of cytochrome c [54], and depolarization of the mitochondrial membrane [43,50,53]. SFN-induced apoptotic cell death was accompanied by the inhibition of IκB (Inhibitor of nuclear factor kappa B) phosphorylation and degradation, and the nuclear translocation of p65, a subunit of nuclear factor kappa B (NF-κB). SFN has been shown to activate the NRF2/KEAP1 (Nuclear factor erythroid 2-related factor 2/Kelch-like ECH-associated protein 1) signaling pathway [38] and to influence ROS production [49,50,53,55] and glutathione (GSH) levels [43,51,56]. Changes in ROS and GSH levels appear to depend on the time point and drug concentration.

SFN induced the cytodifferentiation of HL60 cells toward both the granulocytic and macrophage lineages. This process was mediated by PI3K/PKC (phosphatidylinositol 3-kinase/protein kinase C) [39]. In addition to inducing apoptosis, SFN has been shown to induce non-apoptotic cell death in leukemia, specifically ferroptosis [51] and autophagy [31].

SFN has also demonstrated anti-leukemic activity in in vivo models. In a WEHI-3-induced leukemia BALB/c mouse model, SFN enhanced immune responses by increasing macrophage phagocytic activity and natural killer (NK) cell cytotoxicity, while also promoting T- and B-cell proliferation [57]. Furthermore, in ALL (Acute Lymphoblastic Leukemia) xenograft models established by injecting Nalm-6 cells into NOD/SCID mice, SFN treatment reduced tumor burden and inhibited leukemic cell expansion [42].

#### 4.1.2. AITC

Another GSL, sinigrin, which is found in mustard seeds, horseradish, and wasabi, is converted by myrosinase into the unsaturated aliphatic ITC, AITC [58], which is responsible for the characteristic pungent aroma and flavor of these plants. This compound is often cited for its anticancer and antimicrobial properties [59,60].

The IC_50_ values for AITC in AML (Acute Myeloid Leukemia) and ALL cell lines have been reported to range from 2.5 to 6.8 μM, regardless of the incubation time [27,28,29,31]. In contrast, the LD_50_ value determined in the Jurkat cell line after 24 h of cultivation using propidium iodide (PI) staining was significantly higher at 42 ± 3 μM [30]. This discrepancy likely reflects differences in assay endpoints and methodological variations. While assays based on membrane integrity (e.g., PI staining) primarily detect late apoptosis or necrosis, metabolic or proliferation assays may detect earlier cytostatic effects. This may be consistent with findings that AITC can induce cell cycle arrest; this theory is supported, specifically at the G2/M phase in L1210 and HL-60 cells [28,31] and at the G1 phase in HL-60 cells [27]. However, AITC appears to be less potent at inducing apoptosis than other ITCs [27,28,31]. Furthermore, AITC was shown to induce autophagy in L1210 cells [31], suggesting that its anticancer activity may involve alternative stress-response pathways in addition to apoptosis. It has also been demonstrated that AITC inhibits the proliferation of DS19 mouse erythroleukemia cells and significantly increases histone acetylation [61].

It has been demonstrated that AITC induces apoptosis in leukemia cells via various converging pathways. One such pathway involves activation of ROCK1 (Rho-associated, coiled-coil-containing protein kinase 1), and PTEN (Phosphatase and tensin homolog), subsequent PI3K inhibition and dephosphorylation of cofilin by PP1/PP2A (Protein Phosphatase 1/ Protein Phosphatase 2A). Activated cofilin then translocates to the mitochondria, leading to the release of cytochrome c and triggering intrinsic apoptosis [62]. Another pathway involves JNK (C-Jun N-Terminal Kinases) activation and BID (BH3 Interacting Domain Death Agonist) cleavage into active fragments [63]. Treatment of the cells with AITC activates caspases 3, 9 [27,62], and 8 [27].

#### 4.1.3. BITC

Aromatic BITC is produced by the myrosinase-mediated hydrolysis of glucotropaeolin (also known as benzyl glucosinolate) [64]. Sources of glucotropaeolin include cress (Lepidium sativum), nasturtium (Tropaeolum majus), and certain radishes and mustards. There is evidence that BITC exhibits both anti-cancer and antimicrobial activities [65,66].

In the Jurkat cell line, the IC_50_ for BITC was calculated from AlamarBlue assay data in one study [36], whereas the LD_50_ was determined by PI staining in another study [30]. These values are broadly comparable, although derived from different assay endpoints, which may be consistent with the high apoptotic potential observed for this drug [27,28,30,36]. BITC has been reported to activate multiple stress and apoptotic pathways. Treatment of cells with BITC led to activation of caspase 3 [27,33,67], caspase 9 [27,67], and caspase 8 [27], as well as PARP cleavage [67,68]. The JNK and p38 MAPK (mitogen-activated protein kinases) pathways were activated [36]. It has been shown that BITC induces ROS production and DNA damage [33,35].

It also reduces mitochondrial membrane potential [27,28,33]. Furthermore, BITC induces autophagy, which has been suggested to act as a defense mechanism against BITC-induced apoptosis [32]. BITC has been shown to block the cell cycle at various phases, with G2/M phase arrest being the most common [27,28,36,67]. However, arrests have also been reported in the S phase [32] and the G0/G1 phase [33].

Exposure of Jurkat cells to BITC resulted in the accumulation of the nuclear apoptosis-inducing factor (AIF), the translocation of the Bcl2-associated X protein (Bax), and the downregulation of myeloid cell leukemia-1 (Mcl-1) protein. This effect has been linked to the inhibition of Mcl-1 translation (rather than transcription), potentially through the dephosphorylation of eIF4G (Eukaryotic initiation factor 4 gamma) [67].

BITC and PEITC have also been shown to inhibit deubiquitinating enzymes associated with tumorigenesis, including USP9x (Ubiquitin-Specific Peptidase 9, X-Linked), which protects the anti-apoptotic protein Mcl-1 from degradation. Furthermore, they have been shown to increase the ubiquitination of the oncogenic fusion protein Bcr-Abl, leading to its degradation at low ITC concentrations and aggregation at higher concentrations [34].

Although BITC induced expression of classic apoptotic markers, such as the exposure of phosphatidylserine on the cell surface and mitochondrial membrane depolarization, it failed to promote progression to apoptosis or the final breakdown of U937 cells into apoptotic bodies. BITC-treated cells also released fewer chemoattractants, such as IL-8 (interleukin-8) and MCP-1 (Monocyte Chemoattractant Protein-1), potentially impairing efferocytosis (debris clearance). While many studies report downregulation of Mcl-1 (a survival protein) in BITC-induced apoptosis, this study did not observe Mcl-1 downregulation in U937 cells. Instead, they noted increased levels of BAG-1 (Bcl2-associated athanogene 1) and PUMA (p53 upregulated modulator of apoptosis), two proteins involved in the stress response and the regulation of apoptosis [68]. In vivo, BITC has been shown to reduce the weights of the liver and spleen and enhance the phagocytic activity of macrophages [33].

#### 4.1.4. PEITC

Another aromatic ITC, PEITC, is produced from the GSL gluconasturtiin, also known as phenethyl glucosinolate. This compound is commonly found in watercress (*Nasturtium officinale*) and in certain types of mustard and other cruciferous plants.

PEITC has been shown to have proapoptotic effects on cell models [69]. Treatment of cells with PEITC has been shown to increase the expression or induce activation of caspase-3, -8, and -9 [29,37,70,71], accompanied by PARP cleavage [71]. Furthermore, PEITC has been shown to promote the proteolytic processing of p22 BID into its truncated active fragments [72] and to downregulate the anti-apoptotic protein Mcl-1 [37,71]. Inactivation of Akt (Protein kinase B) and activation of JNK were also observed [34,72].

PEITC has also been reported to induce oxidative stress, characterized by GSH depletion [37,72,73] and increased ROS generation and accumulation [37,70,73].

In vivo studies in WEHI-3 leukemia-bearing mice demonstrate that PEITC enhances macrophage-mediated phagocytosis and increases NK cell activity. It also reduces spleen and liver weights, collectively indicating tumor-suppressive effects [74]. Moreover, PEITC significantly suppressed tumor growth in a U937 xenograft model by inducing apoptosis, accompanied by Akt inactivation, JNK activation, and downregulation of Mcl-1 [71].

The effects of PEITC on the cell cycle of leukemia cells remain poorly understood. One study reported G2/M phase arrest after 24 h of treatment, but no arrest was observed after 6 h [28]. It is unclear whether the apparent lack of cell cycle arrest reflects a true absence of effect or is simply due to the limited scope of existing studies.

#### 4.1.5. 6-MITC

6-MITC is a structural analog of SFN. Like SFN, it contains a methylsulfinyl end group, separated from the -NCS group by a six-carbon chain. 6-MITC is formed by the enzymatic hydrolysis of glucohesperin (6-methylsulfinylhexyl glucosinolate) by myrosinase [75]. Glucohesperin is found in wasabi, Japanese radish (daikon, *Raphanus sativus*), and other cruciferous plants.

Watanabe et al. found that 6-MITC induced apoptosis in U937 cells [76]. Apoptosis was associated with caspase-8 activation. 6-MITC limited tumor growth by slowing and blocking the cell cycle (G1) in Jurkat and HL-60 cells, respectively, in a dose- and time-dependent manner. It also demonstrated the ability to induce the cytodifferentiation of HL-60 cells into macrophage and granulocytic phenotypes [77]. 6-MITC induced autophagy in Jurkat and HL-60 cells, increasing intracellular ROS levels [78]. 6-MITC has been shown to cause mitotic arrest and autophagy in K562 cells, a human chronic myelogenous leukemia (CML) cell line [79].

#### 4.1.6. Comparison of Natural ITCs

Among the most studied ITCs, BITC and PEITC appear to have the strongest effects on inducing apoptosis and dissipating mitochondrial potential. In contrast, AITC seems to have a more pronounced impact on cell cycle arrest. Compared with SFN, all three exhibit faster, less time-dependent activity [27,28]. The IC_50_ values for AITC, BITC, and PEITC after a 3 h treatment (followed by 69 h of incubation without ITC) were very similar (less than 1-fold increase) to those after a 72 h treatment in all cases. In contrast, while the IC_50_ values for SFN with a 72 h treatment were similar to those of the other three ITCs, SFN’s ability to inhibit cell growth was markedly reduced with only a 3 h treatment [27]. However, observed effects are highly dependent on cell type, concentration, and exposure time. Taken together, ITCs, bioactive compounds derived from GLSs, have attracted considerable interest as potential agents for treating cancer and leukemia. Preclinical studies using various leukemia models show that these compounds have pleiotropic antineoplastic effects by modulating multiple cellular pathways.

#### 4.1.7. Role of Autophagy in ITC-Induced Cytotoxicity

One manifestation of the pleiotropic activity of ITCs is the induction of autophagy, a highly context-dependent process that can either support cellular survival or contribute to cell death following ITC exposure. Autophagy represents an adaptive cellular response to various stress conditions, including oxidative stress induced by anticancer agents. In contrast to apoptosis, which invariably culminates in cell death, autophagy primarily functions as a cytoprotective mechanism that promotes cellular survival by degrading and recycling damaged organelles and macromolecules, thereby maintaining cellular homeostasis. However, when cellular damage exceeds the repair capacity of the autophagic machinery or persists for prolonged periods, autophagy may ultimately contribute to cell death [73,80].

Under moderate stress, autophagy generally promotes cell survival by maintaining cellular homeostasis and removing damaged intracellular components. In contrast, sustained oxidative stress or severe cellular damage may shift autophagy toward a pro-death mechanism. This dual role of autophagy has been demonstrated in several cancer models, in which inhibition of SFN-induced autophagy enhanced apoptotic signaling and increased cell death, suggesting a protective role for autophagy under certain conditions [80,81,82].

The apparently conflicting roles of autophagy reported across different ITCs likely reflect differences in leukemia subtype, differentiation status, treatment duration, ITC concentration, and the magnitude of ROS accumulation. In our previous studies using mouse lymphoblastic leukemia L1210 cells, aliphatic ITCs such as AITC and SFN predominantly induced autophagy, whereas apoptosis played only a minor role [31]. Taken together, these findings suggest that ITC-induced autophagy should not be viewed as uniformly pro-survival or pro-death, but rather as a dynamic stress response whose functional outcome depends on the biological and experimental context.

#### 4.1.8. Selectivity of ITCs Toward Leukemia Cells

The selectivity of ITCs toward malignant hematopoietic cells is not fully understood, and the available evidence is sometimes contradictory. Among natural ITCs, SFN has been investigated most extensively; however, its cytotoxic specificity remains unclear. Several studies have shown that SFN affects both malignant and non-malignant lymphocytes. In non-transformed, phytohemagglutinin-stimulated human lymphocytes, SFN arrested the cell cycle in the G1 phase by reducing cyclin D3 expression. It also induced apoptosis and necrosis via p53 upregulation. These results imply that SFN may act as a growth modulator in T cells [44]. Similarly, dose-dependent cytotoxicity has been observed in both differentiated HL-60 cells and normal human lymphocytes, with higher concentrations producing more pronounced effects [38,55].

Studies directly comparing malignant and nonmalignant cells have yielded inconsistent results. Misiewicz et al. (2007) reported greater sensitivity of lymphoblastoid cells than leukemia cells to SFN, despite enhanced induction of quinone reductase activity in lymphoblastoid cells at lower concentrations. [43]. In contrast, Suppipat et al. (2012) found that healthy peripheral blood mononuclear cells (PBMCs) and lymphoblastoid cells exhibited significantly higher IC_50_ values than pre-B ALL and T-ALL cell lines or primary patient samples [42]. This suggests that malignant cells may be more sensitive in certain contexts [42]. These discrepancies may reflect differences in experimental design including exposure time, dosing regimen, cell density, viability assays, and the use of established cell lines versus primary cells. In addition, the biological activity of ITCs is strongly influenced by extracellular conditions, including serum content, cysteine availability, GSH concentration, and other nucleophilic constituents of the culture medium that can affect ITC stability, bioavailability, and cellular uptake. Differences in antioxidant capacity and intracellular detoxification mechanisms may further contribute to variability in cellular responses.

Compared with SFN, the available evidence for BITC and PEITC more consistently supports preferential toxicity toward malignant hematopoietic cells. BITC demonstrated substantially greater cytotoxicity in canine cancer cell lines than in canine PBMCs and was also more potent in HL-60 leukemia cells than in normal lymphocytes [35,83]. Similarly, PEITC exhibited selective cytotoxicity toward CML and CLL cells and induced apoptosis in multiple leukemia cell lines and primary AML blasts while showing markedly lower toxicity toward normal PBMCs [37,71,84]. 6-MITC exerted a stronger cytotoxic effect on leukemic cells than on healthy blood cells [77].

The molecular basis underlying the apparent selectivity of ITCs remains uncertain. One potential explanation is the altered redox state of cancer cells, which often exhibit higher basal ROS levels and a greater dependence on antioxidant systems, including GSH- and NRF2-mediated pathways, to maintain viability. Consequently, further disruption of redox homeostasis by ITCs may preferentially affect malignant cells. In addition, differences in cellular detoxification capacity and stress-response signaling may contribute to the observed differential sensitivity [85]. In addition, thiol modification of critical proteins may selectively affect transformed cells. Supporting this possibility, PEITC, BITC, and SFN preferentially induced nucleosomal DNA fragmentation in Top2α-proficient HL-60 cells compared with DNA topoisomerase IIα (Top2α)-deficient HL-60/MX2 cells [86].

Overall, the available evidence suggests that certain ITCs may exhibit greater cytotoxicity toward malignant hematopoietic cells than toward their normal counterparts. However, the currently available data remain limited and are derived from a relatively small number of studies employing different experimental models and conditions. While the evidence for PEITC and BITC is generally consistent with preferential toxicity toward malignant cells, the number of studies directly comparing malignant and normal hematopoietic cells remains insufficient to draw definitive conclusions. Findings obtained with SFN are even more heterogeneous, with some studies reporting preferential toxicity toward malignant cells and others demonstrating comparable or greater sensitivity of normal lymphoid cells. Consequently, although the current data suggest a potential therapeutic window for certain ITCs, their selectivity for leukemia cells remains incompletely characterized and warrants further investigation.

### 4.2. Biological Activity of Structural Analogs of ITCs in Leukemia

Although neither naturally occurring ITCs nor synthetic ITC derivatives have been extensively studied in leukemia models, several studies have reported notable results.

#### 4.2.1. Heteroaromatic and Sulfur-Oxidation Variants

Shi et al. (2016) [41] synthesized a panel of SFN analogs incorporating furan, methoxypyridine, methoxybenzene, tetrazole, and thiazole moieties. Among these, the tetrazole derivatives **3d** (IC_50_ = 1.52 ± 0.38 μM), **8d** (IC_50_ = 0.51 ± 0.14 μM), and **9d** (IC_50_ = 0.88 ± 0.28 μM) were markedly more potent than SFN (8.24 ± 2.81 μM) in KG-1a cells (MTT assay, 48 h). Among the methoxy-substituted heteroaromatics, sulfide **3c** (6.30 ± 1.45 μM) and sulfoxide **8c** (4.01 ± 0.97 μM) exhibited slightly greater potency than SFN, whereas sulfone **9c** (10.95 ± 2.38 μM) displayed comparable activity. These findings suggest that incorporating heteroaromatic substituents can substantially affect ITC potency in leukemia cells. In particular, the enhanced activity of tetrazole-containing derivatives may be associated with altered electronic properties and improved interactions with intracellular targets. Furthermore, the observation that sulfoxide derivatives were generally more active than the corresponding sulfides or sulfones supports the idea that an intermediate sulfur oxidation state may provide an optimal balance among reactivity, stability, and cellular permeability [41].

#### 4.2.2. Phosphonate Analogues

Psurski et al. (2017) [87] investigated SFN phosphonate analogues in both HL-60 cells and mitoxantrone-resistant HL-60 cells using an MTT assay over 72 h. HL-60 cells were highly sensitive to compounds **45** (0.5 ± 0.2 μM), **51** (2.9 ± 0.1 μM), and **70** (0.8 ± 0.2 μM). Resistant HL-60 cells showed slightly reduced sensitivity to these compounds, with IC_50_ values of **45** (4.0 ± 0.8 μM), **51** (4.0 ± 0.9 μM), and **70** (3.3 ± 0.8 μM). Nevertheless, the IC_50_ values remained low. The IC_50_ of SFN in HL-60 cells was not reported in this study [87]. The high potency of several phosphonate analogues, including against mitoxantrone-resistant HL-60 cells, suggests that these derivatives may partially overcome multidrug resistance mechanisms. Although the precise basis for this effect remains unclear, the phosphonate moiety may influence intracellular retention, metabolic stability, or interactions with redox-sensitive pathways. These observations suggest that phosphonate modification may be a promising strategy for developing active ITCs against resistant leukemia phenotypes.

#### 4.2.3. 2-Oxohexyl ITC and Alyssin

Misiewicz et al. (2007) [43] compared the activity of 2-oxohexyl ITC and alyssin (5-methylsulfinylpentyl ITC) in B-lymphoblastoid and CCRF-SB leukemia cells. Both compounds exhibited IC_50_ values similar to or slightly higher than those of SFN in healthy cells, but lower in CCRF-SB cells. Notably, 2-oxohexyl ITC was a stronger inducer of quinone reductase (QR) in both healthy and leukemic cells, potentially promoting adaptive resistance mechanisms in leukemic cells. In contrast, alyssin did not markedly increase QR activity in leukemic cells [43]. Both SFN and 2-oxohexyl ITC induced growth arrest and apoptosis in L1210 leukemia cells [88]. These findings suggest that even structurally related ITCs may differ substantially in the balance of cytotoxic and cytoprotective effects. Therefore, preserving anticancer activity while minimizing the induction of cytoprotective pathways is an important consideration in designing future ITC derivatives with improved selectivity for leukemic cells.

#### 4.2.4. Sulfur Oxidation State Analogs

Jakubikova et al. (2005) reported that SFN sulfide (erucin) and sulfoxide (iberin) analogs were approximately twice as effective as SFN in HL-60 cells [28].

Fimognari et al. (2004) found that erucin retained Jurkat cells in the G_2_/M phase and triggered apoptosis by upregulating p53 and Bax, without affecting non-transformed T lymphocytes at concentrations of 10 μM or lower [89].

Doudican et al. (2010) [90] demonstrated that the SFN sulfone analog (erysolin) and SFN significantly enhanced Arsenic trioxide (ATO) cytotoxicity in K-562, HL-60, and U-937 cells by inducing mitochondrial-mediated apoptosis and increasing ROS levels. In contrast, erucin was highly effective in K-562 cells, moderately effective in HL-60 cells, and ineffective in U-937 cells for ATO potentiation [90].

Lin et al. (2023) [91] demonstrated that the sulfide (I7447) and sulfonyl (I7557) analogs of 6-MITC exhibit effects comparable to 6-MITC in human CML cells, including G2/M cell cycle arrest, aberrant mitosis, and autophagy induction. The IC_50_ values of 6-MITC, I7447, and I7557 in K562 cells after 48 h were 4.12 µM, 4.00 µM, and 4.96 µM, respectively, whereas in HEL cells they were 1.43 µM, 5.50 µM, and 1.06 µM, respectively. Notably, both 6-MITC and I7557 suppressed the viability of IM-resistant K562 cells, whereas I7447 showed no activity in IM-resistant cells [91]. Taken together, these studies highlight the importance of sulfur oxidation in modulating the biological activity of ITCs. Sulfide, sulfoxide, and sulfone derivatives displayed distinct effects across leukemia models, likely reflecting differences in redox behavior, ROS generation, and intracellular metabolism. The variable ability of erucin to potentiate ATO cytotoxicity across different cell lines further suggests that leukemia subtype-specific metabolic or antioxidant characteristics may strongly influence ITC responsiveness. Moreover, the retained activity of certain derivatives in imatinib-resistant cells indicates their potential utility in disease settings with resistance.

#### 4.2.5. Phenylhexyl Isothiocyanate (PHI)

PHI is a synthetic ITC known for its broad anticancer effects [92], characterized by a benzene ring separated from the NCS group by six CH_2_ groups. It has been extensively investigated as a potential therapeutic agent in leukemia. Its cytotoxicity has been evaluated across various hematological malignancy cell lines using the MTT assay. PHI showed the strongest activity in AML-M2 models, with IC_50_ values of 7.45 ± 0.89 μM in Kasumi-1 cells and 7.86 ± 0.77 μM in SKNO-1 cells, whereas markedly lower sensitivity was observed in Jurkat, MEG-01, MOLT-4, and MV4-11 cells (IC_50_ > 30 μM) [93]. However, flow cytometry-based analysis reported moderate activity in Jurkat cells, with IC_50_ values of 11 ± 0.6 μM in wild-type cells and 25 ± 0.3 μM in Bcl-2-overexpressing cells [30]. These differences likely reflect methodological variation and intrinsic heterogeneity in leukemia subtypes.

Beyond its cytotoxic effects, PHI is particularly notable for its epigenetic activity in leukemia models. It induces G1 cell cycle arrest and apoptosis, accompanied by decreased histone deacetylase (HDAC) expression and activity, as well as increased histone H3 and H4 acetylation [94,95,96]. PHI has also been shown to reverse aberrant histone methylation patterns, induce DNA demethylation in Molt-4 cells, and reactivate silenced tumor suppressor genes such as p15 [94,96,97]. In p53 R248Q-mutated leukemia cells, PHI restores p53 pathway signaling and triggers downstream pro-apoptotic targets, including BAX and p21 [93]. Notably, PHI demonstrates limited toxicity toward normal peripheral blood and bone marrow mononuclear cells, indicating a degree of tumor selectivity [94].

In vivo, PHI inhibits tumor growth and induces apoptosis in HL-60 xenograft models by targeting cell cycle regulators, without detectable toxicity in normal tissues [98]. It also acts synergistically with the PI3K inhibitor LY294002, enhancing apoptosis and reducing HL-60 cell viability [99].

Collectively, PHI represents a distinct class of ITC derivatives in leukemia research due to its pronounced epigenetic activity and tumor selectivity. While naturally occurring ITCs such as SFN, PEITC, and BITC primarily exhibit epigenetic effects in solid tumors [100], comparable mechanisms in hematologic malignancies have not been sufficiently explored. The unique profile of PHI may be partly explained by its elongated hydrophobic phenylhexyl side chain, which could enhance cellular uptake, intracellular retention, and interactions with nuclear epigenetic targets. However, it is unclear whether these effects are truly unique to PHI or simply underreported for other ITCs, underscoring a broader gap in understanding the SAR within this compound class.

#### 4.2.6. Structural Features Influencing Anti-Leukemic Activity

The anti-leukemic activity of synthetic ITC analogs appears to be influenced by both the sulfur oxidation state and the structural features of substituents attached to the electrophilic isothiocyanate pharmacophore (–NCS), which mediates interactions with cellular nucleophiles and redox-sensitive proteins. Intermediate sulfur oxidation states, particularly sulfoxides, often showed greater anti-leukemic activity than the corresponding sulfides or sulfones. This suggests that redox-related properties may influence biological efficacy. Incorporating heteroaromatic moieties, particularly tetrazole-containing substituents, substantially increased the potency of certain compounds in specific leukemia cell lines, potentially due to altered electronic characteristics and improved interactions with intracellular targets. Increased hydrophobicity, as observed in PHI, may facilitate cellular uptake and subcellular localization, thereby contributing to its pronounced epigenetic activity. Notably, several analogs retained activity in drug-resistant leukemia models, suggesting that modifying ITC structures could help overcome resistance-associated mechanisms. Taken together, these findings imply that future ITC derivatives should balance electrophilicity, redox activity, lipophilicity, and selectivity for leukemic cells to maximize therapeutic efficacy while minimizing toxicity.

The chemical structures of the above-mentioned compounds are summarized in Table 2.

### 4.3. Overcoming Therapeutic Resistance in Leukemia Through ITCs

Despite advances in leukemia therapy, resistance and disease relapse continue to limit long-term success. Researchers are exploring novel approaches, including compounds that enhance the efficacy of established chemotherapeutics. In this context, ITCs, which have demonstrated promising activity across various leukemia models, offer a potential strategy to overcome resistance and enhance treatment outcomes.

In addition to direct cytotoxicity, ITCs target regulatory mechanisms that are crucial for therapy resistance. These properties highlight the therapeutic potential of ITCs in treating cancer and leukemia, as well as their capacity to reverse multidrug resistance (MDR)—a feature that is equally important for improving long-term treatment outcomes.

**Figure 2:**
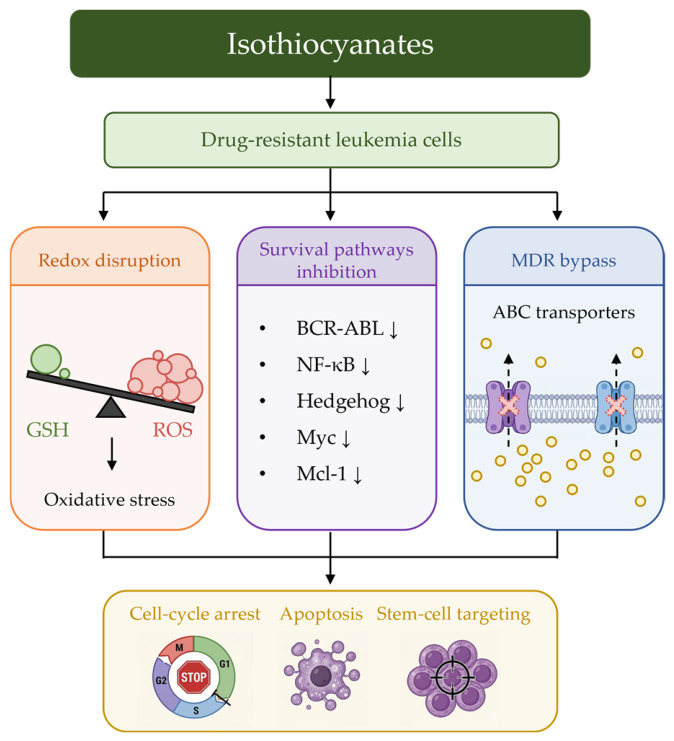
Mechanisms of action of ITCs in overcoming leukemia resistance.

The potential of ITCs to overcome resistance or enhance the effects of other anti-leukemic drugs is further described in this chapter and summarized in Figure 2 and Table 3.

#### 4.3.1. ABC Transporter-Mediated Multidrug Resistance

Multidrug resistance mediated by ATP-binding cassette (ABC) transporters such as P-glycoprotein (P-gp) and multidrug resistance-associated protein 1 (MRP-1), poses a significant obstacle to chemotherapy. It appears that ITCs can overcome this resistance.

For instance, doxorubicin-resistant HL60 cells overexpressing MRP-1 showed comparable sensitivity to AITC, BITC, PEITC, and SFN to their parental cells [27]. Similarly, vincristine-resistant SKM-1 cells overexpressing P-gp responded to SFN and BITC with IC_50_ values close to those of the parental cell line [32], while P-gp-overexpressing L1210 cells showed similar sensitivity to SFN and AITC compared to controls [31]. A systematic comparison of six dietary ITCs (AITC, BITC, PEITC, SFN, ERN and IBN) in multidrug-resistant HL60/ADR (MRP-1^+^) and HL60/VCR (P-gp^+^) cells revealed modest increases in IC_50_ values (most ITCs showed a 2-5-fold increase; SFN showed a ~1.5-fold increase). Despite this reduced sensitivity, BITC and PEITC remained highly potent (IC_50_ < 4 μM), and all tested ITCs induced time- and dose-dependent G_2_/M arrest and apoptosis in the resistant cells [28].

Although these findings suggest that ITCs can partially overcome ABC transporter-mediated drug resistance, the precise interactions between individual ITCs and specific transporters remain incompletely understood. Available evidence indicates that some ITCs may themselves be substrates of ABC transporters. Sulforaphane has been proposed to undergo efflux via P-gp and MRP1 [101], whereas aromatic ITCs such as PEITC and α-naphthyl isothiocyanate appear to be transported predominantly by MRP family members in a GSH-dependent manner [102]. Similarly, pharmacological inhibition of MRPs with MK571 enhanced intracellular accumulation of BITC and potentiated its antiproliferative effects, suggesting that MRP-mediated efflux may limit the intracellular activity of certain ITCs. However, the specific transporters responsible for BITC export have not yet been conclusively identified, although MRP2 has been proposed as a likely candidate [103]. In addition, ITCs readily conjugate with intracellular GSH, which may contribute to their ability to overcome multidrug resistance. By depleting the GSH pool, ITCs could reduce the formation and export of GSH-dependent drug conjugates via MRP transporters, thereby increasing intracellular retention of chemotherapeutic agents. Furthermore, ITC–GSH conjugates themselves may compete with other transporter substrates for efflux, potentially further limiting drug export.

These observations highlight the need for further studies to determine whether individual ITCs act as substrates, inhibitors, or modulators of specific ABC transporters. Such knowledge may have important therapeutic implications, as ITCs could potentially be combined with conventional chemotherapeutic agents that are ABC transporter substrates to increase intracellular drug retention and restore chemosensitivity in resistant leukemia cells. In particular, future studies should evaluate whether the modulation of ABC transporter activity and GSH-dependent drug efflux by ITCs can be exploited to enhance the efficacy of conventional chemotherapy in resistant leukemia models.

#### 4.3.2. Resistance in CML

Chronic myeloid leukemia (CML) accounts for around 20% of adult leukemias. It is characterized by the presence of the Philadelphia chromosome, which results from a reciprocal translocation between chromosomes 9 and 22. This abnormality creates the BCR-ABL fusion gene, which encodes a constitutively active tyrosine kinase that drives the uncontrolled proliferation and survival of leukemic cells. The introduction of tyrosine kinase inhibitors (TKIs), such as imatinib (IM), has revolutionized CML treatment by specifically targeting BCR-ABL. However, resistance to TKIs remains a challenge, which is often, but not always, due to point mutations in the BCR-ABL kinase domain [84].

Recent research has shown that ITCs may help to overcome both mutation-dependent and -independent forms of TKI resistance.

PEITC has been shown to inhibit crosstalk between BCR-ABL and PKC signaling, thereby increasing the sensitivity of CML cells to IM [84]. Furthermore, BITC and PEITC were found to increase the ubiquitination of the BCR-ABL protein, resulting in its degradation at low concentrations and aggregation at higher concentrations. These effects appear to correlate with inhibition of USP9x, a deubiquitinase suggested to stabilize BCR-ABL, as USP9x silencing alone has been shown to reduce BCR-ABL levels [34].

Importantly, PEITC exhibited cytotoxicity in IM-resistant CML cells, regardless of BCR-ABL mutation status, by inducing ROS-mediated mitochondrial damage [104,105].

6-MITC did not alter the BCR-ABL protein expression in IM-resistant K562R cells [79]; however, it suppressed cell viability, an effect also observed with its synthetic analogue I7557 [91].

Combined treatment with IM and SFN has been shown to effectively eliminate CD34^+^/CD38^−^ leukemia stem cells (LSCs) isolated from the CML cell line KU812. These LSCs exhibited elevated BCR-ABL and β-catenin expression and resistance to IM. Combined treatment with IM and SFN re-sensitized LSCs by inducing intracellular ROS, which is an important finding given the role of LSCs in disease relapse [106].

#### 4.3.3. Resistance in AML

In AML, ITCs have demonstrated the ability to overcome several resistance mechanisms. A sub-toxic dose of SFN, when combined with tumor necrosis factor-α (TNF-α), significantly triggered apoptosis in TNF-α-resistant AML and CML cells. SFN suppressed TNF-α-induced NF-κB activity by inhibiting IκBα degradation, thereby inducing apoptosis via ROS-dependent activation of caspase-3 [107].

LSCs are thought to underlie relapse in AML due to their ability to self-renew, proliferate, and enter a state of quiescence. Aberrant activation of the Sonic Hedgehog (Shh) signaling pathway has been associated with promoting leukemogenesis and maintaining LSC survival. It has been shown that SFN preferentially suppresses proliferation in Shh-overexpressed AML cells compared to Shh-downregulated cells, suggesting that it can interfere with Hedgehog-driven stemness [40].

ITCs can also act synergistically with conventional chemotherapy. For example, BITC has been shown to enhance the cisplatin-induced cytotoxicity in HL-60 cells, but not in normal human lymphocytes, through mechanisms involving ROS generation, GSH depletion, and ERK (Extracellular Signal-Regulated Kinase) signaling [83]. Similarly, PEITC enhances the cytotoxic activity of suberoylanilide hydroxamic acid (SAHA, vorinostat) in AML cell lines and primary leukemia cells, an effect attributed to GSH depletion and enhanced ROS stress [108].

ATO is an effective treatment for acute promyelocytic leukemia (APL), inducing ROS-mediated apoptosis via detoxification systems. However, higher GSH levels are associated with resistance to ATO [109]. SFN increases the sensitivity of leukemia cells to ATO by reducing their intracellular GSH levels. This lowers the cell’s oxidative threshold, making it more susceptible to the cytotoxic effect of ATO [90].

#### 4.3.4. Resistance in CLL

Chronic lymphocytic leukemia (CLL), the most common form of leukemia in adults, is often treated with fludarabine, a purine analog chemotherapy. However, resistance and microenvironmental protection remain major obstacles.

PEITC has demonstrated potent activity against both fludarabine-sensitive and -resistant primary CLL cells, while exhibiting significantly lower toxicity toward normal lymphocytes. This selective cytotoxicity is attributed to the intrinsic redox imbalance in CLL cells, characterized by elevated basal ROS levels and reduced intracellular GSH. This renders CLL cells particularly vulnerable to further oxidative stress. PEITC induces robust ROS accumulation, leading to Mcl-1 degradation and apoptotic cell death. PEITC retains its efficacy in CLL cells lacking functional p53 and in the presence of stromal support. Both of these factors are associated with poor clinical outcomes [37,110].

Furthermore, when combined with the histone deacetylase inhibitor SAHA, PEITC effectively depletes stromal-induced GSH, further enhancing oxidative stress and significantly amplifying apoptosis in CLL cells [111].

In addition to causing redox-mediated cytotoxicity, PEITC has been shown to interfere with translation and signaling pathways critical for CLL survival. In anti-IgM-stimulated CLL cells, PEITC increased eIF2α phosphorylation and inhibited mTORC1 (mechanistic target of rapamycin complex 1), thereby reducing both global translation and translation of specific mRNA (including MYC). Combined treatment with PEITC and the BTK inhibitor ibrutinib further suppressed translation and enhanced cell death. The inhibition of protein synthesis, particularly of MYC, may significantly contribute to the compound’s chemopreventive and therapeutic effects [112].

#### 4.3.5. Resistance in ALL

SFN and PEITC significantly reduced viability and induced apoptosis in camptothecin-resistant and multidrug-resistant T-cell lymphoblastic leukemia CEM/C2 cells in a dose- and time-dependent manner, with IC_50_ values of 22 and 17 μM for SFN, and 12 and 7.8 μM for PEITC after 24 and 48 h, respectively. These findings demonstrate the potent cytotoxic activity of PEITC, and to a lesser extent SFN, against drug-resistant ALL cells [54].

Genetically modifying the Jurkat T-ALL cell line to overexpress the antiapoptotic protein Bcl-2 renders it resistant to cytarabine (AraC), etoposide, and melphalan. However, PEITC and BITC effectively induce apoptosis in these Bcl-2-overexpressing cells [30].

**Table 3:** ITCs in Overcoming Leukemia Resistance and Chemotherapy Enhancement.

Leukemia Model	Resistance	ITC	Effect	Reference
**CML**				
K562, KU812	-	PEITC (+imatinib)	Inhibited Bcr-Abl and PKC crosstalk, increased efficacy of imatinib	[84]
KBM5, BaF3, 32D, patients	Imatinib (T315I BCR-ABL mutation)	PEITC	GSH depletion and ROS-induced BCR-ABL degradation	[104]
DA1-3b, K562, patients	Imatinib (w/wo BCR-ABL mutation)	PEITC	ROS-mediated cytotoxicity	[105]
KU812	Imatinib (LSCs)	SFN (+imatinib)	ROS-mediated apoptosis, re-sensitized LSCs; reduced BCR-ABL	[106]
**CML/AML**				
THP-1, HL60, U937, K562	TNF-α	SFN (+TNF-α)	Suppressed TNF-α-induced NF-κB activity; ROS	[107]
HL-60, K-562, U-937	-	SFN (+ATO)	GSH depletion; ROS	[90]
**AML**				
KG1, KG1a	LSCs (Shh overexpression)	SFN	Hedgehog signaling suppression	[40]
HL60	-	BITC (+cisplatin)	ROS; GSH depletion; ERK signaling	[83]
HL60	Doxorubicin (MRP-1)	AITC, BITC, PEITC, SFN	Similar IC_50_ to parental cells	[27]
HL60	Doxorubicin (MRP-1) Vincristine (P-gp)	AITC, BITC, PEITC, SFN, ERN, IBN	Modest IC_50_ increase; G2/M arrest, apoptosis	[28]
SKM-1	Vincristine (P-gp)	SFN, BITC	Similar IC_50_ to parental cells	[32]
HL60, U937, ML1, patients	Pan-HDACi	PEITC (+SAHA)	GSH depletion; ROS	[108]
**CLL**				
patients	Microenvironment-mediated	PEITC (+SAHA)	GSH depletion; ROS; Mcl-1 downregulation; cardiolipin oxidation	[111]
patients	Microenvironment + p53 deficiency	PEITC	GSH depletion, ROS; Mcl-1 downregulation	[110]
patients	Fludarabine	PEITC	GSH depletion, ROS, Mcl-1 downregulation, cardiolipin oxidation	[37]
patients	-	PEITC (+ibrutinib)	blocked translation (MYC), enhanced cytotoxicity	[112]
**ALL**				
Jurkat	AraC, etoposide, melphalan (Bcl-2 overexpression)	PEITC, BITC	Induced apoptosis	[30]
CEM/C2	Camptothecin; MDR	SFN, PEITC	Induced apoptosis	[54]
L1210	P-gp	SFN, AITC	Similar IC_50_ to parental cells	[31]

## 5. Future Directions

Despite promising preclinical evidence supporting the anticancer potential of ITCs in leukemia, there are still several gaps that require further investigation. Future research should focus on validating the observed cytotoxic and pro-apoptotic effects in vivo, with an emphasis on evaluating selectivity toward leukemic versus normal hematopoietic cells. Optimizing dosage, pharmacokinetics, and delivery strategies is essential for translating ITCs into clinical applications. Structural modifications and synthetic analogs offer opportunities to enhance potency, selectivity, and bioavailability; however, their mechanistic profiles and safety must be rigorously characterized. In addition to pathway-specific effects, it is important to consider the inherently reactive nature of ITCs and their potential for off-target interactions. Due to the electrophilic character of the –NCS group, ITCs can readily react with nucleophilic residues, particularly cysteine thiols, across a broad spectrum of cellular proteins. Consequently, their biological activity is not restricted to a limited number of defined molecular targets but rather reflects a broader pattern of covalent modifications affecting multiple regulatory proteins and signaling nodes. While this promiscuous reactivity may contribute to the pleiotropic anticancer effects of ITCs, it also complicates the identification of primary molecular targets and may limit their development as highly selective therapeutic agents [113].

Emerging evidence suggests that ITCs modulate epigenetics, including DNA demethylation, histone acetylation, and p53 reactivation. This opens new possibilities for combinatorial therapies. Future studies could explore the synergistic effects of combining ITCs with existing chemotherapeutics, targeted inhibitors, or immunotherapies to potentially overcome drug resistance in leukemia. Future studies could explore the synergistic effects of combining ITCs with existing chemotherapeutics, targeted inhibitors, or immunotherapies to potentially overcome drug resistance in leukemia. Importantly, a major translational limitation is the discrepancy between the micromolar concentrations commonly effective in preclinical models and the comparatively lower systemic exposure reported in human studies, which is influenced by limited bioavailability and rapid metabolism. This issue should be carefully considered when interpreting preclinical efficacy data and designing future translational studies [114].

However, despite these promising findings, clinical evidence in patients with hematological malignancies is currently scarce. To date, no published clinical trials have specifically evaluated ITCs as therapeutic agents in leukemia, lymphoma, or multiple myeloma patients. Most human studies have focused on the safety, pharmacokinetics, and chemopreventive potential of SFN-containing preparations in healthy volunteers or in patients with solid tumors rather than hematological cancers. Early-phase clinical studies have demonstrated that SFN and related broccoli sprout-derived ITCs are generally well tolerated and exhibit favorable safety profiles in humans [115]. Furthermore, a recent systematic review of randomized controlled trials concluded that although SFN shows therapeutic promise in several cancer types, the available clinical evidence remains limited and heterogeneous, underscoring the need for more robust clinical investigations [116].

Taken together, the substantial gap between preclinical efficacy and clinical validation highlights the need for well-designed early-phase clinical trials to determine whether the promising antitumor effects of ITCs observed in experimental models can be translated into meaningful therapeutic benefits for patients with hematological malignancies [117].

## 6. Conclusions

ITCs are naturally occurring compounds derived from GSLs found in cruciferous vegetables. They represent a promising group of bioactive agents with significant chemopreventive and therapeutic potential against leukemia. SFN, PEITC, and BITC are among the most extensively studied compounds and have demonstrated significant antileukemic activity in various experimental models, while AITC and 6-MITC have also shown promise. Together, these compounds exhibit cytotoxic, pro-apoptotic, and differentiation-inducing effects, often selectively targeting malignant cells. The antileukemic activity of ITCs is mediated through multiple interconnected mechanisms, including cell cycle arrest, induction of apoptosis through mitochondrial and caspase-dependent pathways, modulation of ROS/GSH homeostasis, activation of NRF2/KEAP1 signaling, and inhibition of pro-survival pathways such as PI3K/Akt and NF-κB. BITC and PEITC generally exhibit the strongest proapoptotic effects, while AITC primarily exerts cytostatic activity by arresting the cell cycle arrest. SFN demonstrates particularly pleiotropic and context-dependent effects, including the induction of apoptosis, autophagy, and other forms of regulated cell death. Furthermore, synthetic derivatives and structural modifications of natural ITCs have revealed important SARs and, in some cases, enhanced biological potency and selectivity. Emerging evidence also suggests that ITCs can influence epigenetic regulation through mechanisms such as histone modification, DNA demethylation, and restoration of tumor suppressor activity.

Despite these promising findings, several important challenges remain. Most available evidence is derived from in vitro studies. In vivo efficacy, pharmacokinetic characterization, and long-term safety data remain limited. Additionally, the concentrations necessary to produce biological effects in experimental systems may not always be achievable in vivo due to limited bioavailability and rapid metabolism. While several studies suggest preferential activity against malignant cells, the degree of therapeutic selectivity is poorly characterized and requires further investigation.

Overall, ITCs are multifunctional anticancer agents with the potential to serve as standalone therapies or as sensitizers in combination with conventional anticancer drugs. Future research should focus on improving formulation strategies and delivery approaches, identifying rational combination regimens, clarifying molecular targets and selectivity, and conducting well-designed in vivo and early-phase clinical studies. Addressing these challenges will be essential for translating the promising preclinical activity of ITCs into clinically meaningful therapeutic applications for leukemia patients.

## Figures and Tables

**Table 1:** IC_50_ values of natural ITCs in leukemia cell lines and primary cells.

Cell Lines/Primary Cells	IC_50_ [μM]	Exposure Time	Method	Reference
**AITC**				
HL-60 (AML)	3.3 ± 1.1	3 h + 69 h	MTT	[27]
	2.5 ± 0.1	72 h		
HL-60 (AML)	4.0 ± 1.7	72 h	MTT	[28]
HL-60 (AML)	2.56 ± 0.11	48 h	Trypan blue	[29]
ML-1 (AML)	2.59 ± 0.12	48 h	Trypan blue	[29]
Jurkat (T-ALL)	42 ± 3	24 h	FC (PI)	[30]
L1210 (mouse, pre-B ALL)	6.8 ± 1.68	24 h	CASY	[31]
**BITC**				
HL-60 (AML)	2.0 ± 0.3	3 h + 69 h	MTT	[27]
	1.8 ± 2.3	72 h		
HL-60 (AML)	0.6 ± 1.1	72 h	MTT	[28]
SKM-1 (AML)	4.15 ± 0.06	24 h	MTS	[32]
WEHI-3 (mouse, AML)	0.738	24 h	FC (PI)	[33]
K562 (CML)	1–3	48 h	CellTiterGlo	[34]
CLB-70 (canine CML)	3.78 ± 0.25	24 h	FC (PI)	[35]
GL-1 (canine B-cell leukemia)	11.14 ± 1.31	24 h	FC (PI)	[35]
Jurkat (T-ALL)	6	24 h	AlamarBlue	[36]
Jurkat (T-ALL)	6.1 ± 0.1	24 h	FC (PI)	[30]
**PEITC**				
HL-60 (AML)	4.0 ± 1.3	3 h + 69 h	MTT	[27]
	3.6 ± 0.4	72 h		
HL-60 (AML)	1.0 ± 1.9	72 h	MTT	[28]
HL-60 (AML)	1.49 ± 0.01	48 h	Trypan blue	[29]
ML-1 (AML)	2.67 ± 0.06	48 h	Trypan blue	[29]
Jurkat (T-ALL)	7.4 ± 0.1	24 h	FC (PI)	[30]
K562 (CML)	1–3	48 h	CellTiterGlo	[34]
Primary CLL cells	3.5–8.2	72 h	MTT	[37]
Primary CLL cells	4.8	30 h	MTT	[37]
Normal lymphocytes	27	30 h	MTT	[37]
**SFN**				
HL-60 (AML)	31.5 ± 1.3	3 h + 69 h	MTT	[27]
	3.4 ± 0.6	72 h		
HL-60 (AML)	5.1 ± 1.3	72 h	MTT	[28]
HL-60 (AML)	5.9 ± 0.3	48 h	MTT	[38]
	11.3 ± 0.5	72 h		
HL-60 (AML)	49.5	30h	Trypan blue	[39]
SKM-1 (AML)	7.31 ± 0.09	24 h	MTS	[32]
KG1 (AML, LSC model)	9.379	24 h	CCK-8 assay	[40]
	6.928	48 h		
	4.493	72 h		
KG1a (AML, LSC model)	16.89	24 h	CCK-8 assay	[40]
	8.426	48 h		
	7.461	72 h		
KG1a (AML, LSC model)	8.24 ± 2.81	48 h	MTT	[41]
DND41 (T-ALL)	13.49 (12.84–14.15)	24 h	CellTiter-Glo	[42]
	7.14 (6.27–8.01)	48 h		
Jurkat (T-ALL)	4.72 (4.19–5.25)	24 h	CellTiter-Glo	[42]
	2.39 (1.44–3.34)	48 h		
Jurkat (T-ALL)	43 ± 3	24 h	FC (PI)	[30]
KOPT-K1 (T-ALL)	3.04 (2.16–3.92)	24 h	CellTiter-Glo	[42]
	1.24 (0.84–1.66)	48 h		
RPMI (T-ALL)	7.87 (6.40–9.34)	24 h	CellTiter-Glo	[42]
	5.72 (4.12–7.32)	48 h		
Primary T-ALL	10.1 ± 2.0	48 h	CellTiter-Glo	[42]
CCRF-SB (B-ALL)	7.75 ± 0.25	48h	MTT	[43]
L1210 (mouse, pre-B ALL)	10.3 ± 0.45	24 h	CASY	[31]
Nalm-6 (Pre-B ALL)	8.91 (7.43–10.83)	24 h	CellTiter-Glo	[42]
	4.58 (4.37–4.79)	48 h		
REH (Pre-B ALL)	10.26 (9.40–11.20)	24 h	CellTiter-Glo	[42]
	4.38 (4.10–4.67)	48 h		
RS-4 (B-ALL)	9.86 (9.35–10.41)	24 h	CellTiter-Glo	[42]
	3.85 (3.69–4.01)	48 h		
Primary pre-B ALL	13.01 ± 8.1	48 h	CellTiter-Glo	[42]
B-lymphoblastoid (immortalized B-lymphocytes from a healthy person)	5.9 ± 0.16	48h	MTT	[43]
LCL (normal)	80.81 (26.80–243.60)	24 h	CellTiter-Glo	[42]
	30.83 (17.67–53.80)	48 h		
PBMC	30.2 ± 7.2	48 h	CellTiter-Glo	[42]
PHA-stimulated T-cells	~33	48 h	Trypan blue	[44]

IC_50_ values are reported as mean ± SD, or as 95% confidence interval values in parentheses, or as an interval of IC_50_ for several samples. FC—flow cytometry.

**Table 2:** Natural ITCs and Synthetic Analogs studied in Leukemia.

		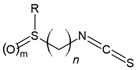			
**Compound**		**Name**	**R**	**n**	**m**
SFN		4-(Methylsulfinyl)butyl isothiocyanate	Methyl	4	1
alyssin		5-(Methylsulfinyl)pentyl isothiocyanate	Methyl	5	1
erucin		4-(Methylthio)butyl isothiocyanate	Methyl	4	0
erysolin		4-(Methylsulfonyl)butyl isothiocyanate	Methyl	4	2
iberin		3-(Methylsulfinyl)propyl isothiocyanate	Methyl	3	1
6-MITC		6-(Methylsulfinyl)hexyl isothiocyanate	Methyl	6	1
I7447	[91]	6-(Methylthio)hexyl isothiocyanate	Methyl	6	0
I7557		6-(Methylsulfonyl)hexyl isothiocyanate	Methyl	6	2
**3d**	[41]	1-((4-Isothiocyanatobutyl)thio)-5-phenyl-1H-tetrazole	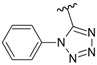	4	0
**8d**		1-((4-Isothiocyanatobutyl)sulfinyl)-5-phenyl-1H-tetrazol		4	1
**9d**		1-((4-Isothiocyanatobutyl)sulfonyl)-5-phenyl-1H-tetrazole		4	2
**3c**	[41]	2-((4-Isothiocyanatobutyl)thio)-6-methoxy-1H-benzo[d]imidazole	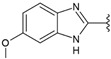	4	0
**8c**		2-((4-Isothiocyanatobutyl)sulfinyl)-6-methoxy-1H-benzo[d]imidazol		4	1
**9c**		2-((4-Isothiocyanatobutyl)sulfonyl)-6-methoxy-1H-benzo[d]imidazole		4	2
		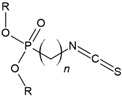			
**Compound**		**Name**	**R**	**n**	
**45**	[87]	Diethyl 6-(isothiocyanato)hexylphosphonate	Ethyl	6	
**51**		Diisopropyl 6-(isothiocyanato)hexylphosphonate	Isopropyl	6	
**70**		Diphenyl 6-(isothiocyanato)hexylphosphonate	Phenyl	6	
		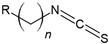			
**Compound**		**Name**	**R**	**n**	
PEITC		Phenylethyl isothiocyanate	Phenyl	2	
BITC		Benzyl isothiocyanate	Phenyl	1	
AITC		Allyl isothiocyanate	Vinyl	1	
PHI		Phenylhexyl isothiocyanate	Phenyl	6	
2-oxohexyl ITC		2-oxohexyl isothiocyanate	Acetyl	4	

Highlighted rows indicate natural ITCs.

## Data Availability

No new data were created or analyzed in this study. Data sharing is not applicable to this article.

## Abbreviations

The following abbreviations are used in this manuscript:

6-MITC	6-methylsulfinylhexyl isothiocyanate
ABC	ATP-binding cassette
AIF	Nuclear apoptosis-inducing factor
AITC	Allyl isothiocyanate
ALL	Acute lymphoblastic leukemia
AML	Acute myeloid leukemia
APL	Acute promyelocytic leukemia
ATO	Arsenic trioxide
BAG-1	Bcl2-associated athanogene 1
BAX	Bcl2-associated X protein
Bcl-2	B-cell lymphoma 2
BCR	Breakpoint cluster region
BID	BH3 Interacting Domain Death Agonist
BITC	Benzyl isothiocyanate
CLL	Chronic lymphocytic leukemia
CML	Chronic myeloid leukemia
elF4G	Eukaryotic initiation factor 4 gamma
FC	Flow cytometry
FITC	Fluorescein isothiocyanate
GSH	Glutathione
GSL	Glucosinolate
HDAC	Histone deacetylase
IκB	Inhibitor of nuclear factor kappa B
IL-8	Interleukin 8
IM	Imatinib
ITCs	Isothiocyanates
JNK	Jun N-terminal kinase
Keap1	Kelch-like ECH-associated protein 1
LSCs	Leukemia stem cells
Mcl-1	Myeloid cell leukemia-1
MCP-1	Monocyte chemoattractant protein-1
MDR	Multidrug resistance
MRP-1	Multidrug resistance-associated protein 1
MTS	3-(4,5-dimethylthiazol-2-yl)-5-(3-carboxymethoxyphenyl)-2-(4-sulfophenyl)-2H-tetrazolium
MTT	3-(4,5-dimethylthiazol-2-yl)-2,5-diphenyltetrazolium bromide
-NCS	ITC functional group
NF-κB	p65-nuclear factor kappa B
-NH2	Amino group
NK	Natural killer
NRF2	Nuclear factor erythroid 2-related factor 2
-OH	Hydroxyl group
PARP	Poly (ADP-ribose) polymerase
PBMCs	Peripheral blood mononuclear cells
PEITC	Phenethyl isothiocyanate
P-gp	P-glycoprotein
PHI	Phenylhexyl isothiocyanate
PI	Propidium iodide
PI3K	Phosphatidylinositol 3-kinase
PKC	Protein kinase C
PSA	Prostate-specific antigen
PTEN	Phosphatase and tensin homolog
PUMA	p53 upregulated modulator of apoptosis
QR	Quinone reductase
ROCK1	Rho-associated coiled-coil containing protein kinase 1
ROS	Reactive oxygen species
SAHA	Suberoylanilide hydroxamic acid
SAR	Structure-activity relationship
SFN	Sulforaphane
-SH	Thiol group
Shh	Sonic hedgehog
TKIs	Tyrosine kinase inhibitors
TNF-α	Tumor necrosis factor-α
USP9X	Ubiquitin-specific peptide 9 X linked
